# Women with prolonged nausea in pregnancy have increased risk for depressive symptoms postpartum

**DOI:** 10.1038/s41598-018-33197-1

**Published:** 2018-10-25

**Authors:** Stavros I. Iliadis, Cathrine Axfors, Sara Johansson, Alkistis Skalkidou, Ajlana Mulic-Lutvica

**Affiliations:** Department of Women’s and Children’s Health, Uppsala University, 751 85 Uppsala, Sweden

## Abstract

The aim of this population-based, longitudinal study was to assess the association between nausea and vomiting in pregnancy (NVP) and perinatal depressive symptoms. Pregnant women (N = 4239) undergoing routine ultrasound at gestational week (GW) 17 self-reported on NVP and were divided into those without nausea (G0), early (≤17 GW) nausea without medication (G1), early nausea with medication (G2), and prolonged (>17 GW) nausea (G3). The Edinburgh Postnatal Depression Scale at GW 17 and 32 (cut-off ≥13) and at six weeks postpartum (cut-off ≥12) was used to assess depressive symptoms. Main outcome measures were depressive symptoms at GW 32 and at six weeks postpartum. NVP was experienced by 80.7%. The unadjusted logistic regression showed a positive association between all three nausea groups and depressive symptoms at all time-points. After adjustment, significant associations with postpartum depressive symptoms remained for G3, compared to G0 (aOR = 1.66; 95% CI 1.1–2.52). After excluding women with history of depression, only the G3 group was at higher odds for postpartum depressive symptoms (aOR = 2.26; 95% CI 1.04–4.92). In conclusion, women with prolonged nausea have increased risk of depressive symptoms at six weeks postpartum, regardless of history of depression.

## Introduction

Nausea and vomiting in pregnancy (NVP) has for a long time fascinated the scientific community for two main reasons: its high prevalence, which has rendered it into one of the symptoms of early pregnancy and its great symptom variability, from early physiological nausea of pregnancy to a more severe condition, which may result even in maternal death at its worst form^1^. NVP affects 50–90% of pregnant women^2–4^. Symptoms begin early in the first trimester, peak at around nine gestational weeks (GW) and typically cease at GW 20^4^. In 0.3–2.3% of cases it progresses to the more severe condition hyperemesis gravidarum (HG)^4,5^ and in 5–22% of affected women the symptoms persist throughout pregnancy^4,6–8^. WHO defines HG as NVP starting before 22 GW but the duration of symptoms and the time-point of symptom ceasing are not noted^9^. The vast majority of published studies focus on HG, the most severe form of NVP requiring hospitalisation and/or parenteral nutrition. However, Chandra *et al*. showed in the Mother Risk Program in Toronto a discordance between physical symptoms and perception of severity among women with NVP^10^. Moreover, less is known about the prolonged form of NVP extending in the second and third trimester of pregnancy.

Previous studies have investigated a possible association between NVP and perinatal depressive symptoms^11,12^. A systematic review and meta-analysis by Mitchell-Jones *et al*., that found increased rates of antenatal depression (AND) in women with HG, points out several methodological flaws, such as heterogeneity of the studies, small sample size, inconsistent definitions of HG, a variety of different and not validated scales for depression assessment, and the lack of adjusting for confounding factors. As a result, only one of 12 studies included in the meta-analysis had low risk of bias^13^.

Although the association between NVP and depression has been extensively studied, the literature from large, population-based studies considering the various spectrum of the disease is scarce. Moreover, few studies have taken into account the history of psychiatric disorders^12^ and their results are inconsistent^11,12,14,15^. A recent large population-based study on the risk of emotional distress during and after pregnancy, including 92 947 pregnant women, showed that women with HG, defined as prolonged NVP requiring hospitalization before the 25^th^ gestational week, were more likely to report emotional distress during pregnancy and at six months postpartum, but not at 18 months postpartum compared to women without HG^16^. Similarly, a large population-based cohort study using Danish registers (N = 392 458) that included only women with HG and without psychiatric history, demonstrated that HG was associated with almost three times increased risk for first time onset postpartum depression (PPD) within one year after delivery^17^. On the other hand, a review of 38 studies without severe forms of HG, explored the impact of nausea on quality of life and demonstrated that nausea was more debilitating than vomiting, recommending caregivers to pay attention even to nausea as a single symptom^18^. Hence, more studies focusing on the association between perinatal depression and various types of NVP, in terms of disease onset and cessation of symptoms, are needed.

The aim of this study was to longitudinally investigate a possible association between different types of NVP within the broad disease spectrum, based on cessation of NVP symptoms and the use of medication, and perinatal depressive symptoms, taking into account the role of previous depression and adjusting for known confounding factors.

## Methods

This study is part of an ongoing longitudinal cohort study of affective disorders during pregnancy and the postpartum period, the BASIC-study (Biology, Affection, Stress, Imaging and Cognition)^19,20^. The BASIC-study is conducted at the Department of Obstetrics and Gynaecology at Uppsala University Hospital, which is a tertiary referral centre responsible for all pregnant women within the county as well as for high-risk pregnancies from nearby counties, including around 4300 deliveries a year. The study has been approved by the Regional Ethical Review Board in Uppsala and all participants provided written informed consent.

All pregnant Swedish-speaking women of at least 18 years of age, who were scheduled for routine ultrasound (around GW 17) between January 2010 and September 2016, were invited to participate in the study. Exclusion criteria were (1) women whose personal data were kept confidential, and (2) women with pathologic pregnancies as diagnosed by the routine ultrasound. In the BASIC project, the participation rate is approximately 22%^21^.

Study subjects were invited to fill-out several web-based questionnaires to assess the main exposure of the study (NVP), the main outcome (self-reported depressive symptoms) as well as several background characteristics. The first survey was answered at enrolment at GW 17 and follow-ups were answered at GW 32 and at postpartum week six. Self-reported experience of nausea in current pregnancy was assessed by three questions at GW 17: “Have you experienced any nausea during this pregnancy?” (Yes or no), “At which gestational week did the nausea cease?” (Text answer), and “Did you receive any medication against nausea in pregnancy?” (Yes or no). Questions on NVP referred to pregnancy-related nausea. No additional question on NVP symptoms was included in the questionnaire at GW 32. A small number of women were recruited before undergoing an elective cesarean delivery, around gestational week 38–39, and filled out the question on nausea at that time-point. Moreover, a few women sent in the questionnaire a few weeks later (the vast majority of them within two weeks after GW 17) and reported at that time-point information about the gestational week at which nausea symptoms ceased. Women were divided into four groups based on their answers: those who did not experience any nausea (controls; group 0, G0); those who experienced nausea ≤17 GW but did not take any medication (early nausea without medication, G1); those who experienced nausea ≤17 GW and used medication (early nausea with medication, G2); and those who experienced nausea >17 GW, regardless of the use of medication (prolonged nausea, G3).

The Edinburgh Postnatal Depression Scale (EPDS)^22^ was administered at all three time points. EPDS is an internationally used screening tool to identify depressive symptoms in the perinatal period. The EPDS consists of ten questions regarding symptoms of depressed mood across the past seven days and items are answered on a four-point scale yielding total scores between 0–30. For the present study, the cut-off used in order to classify participants by depression status, marking clinically significant depressive symptoms, was set at ≥13 points in pregnancy^23^ and ≥12 points postpartum^24^, according to previous Swedish validation studies.

Information was also gathered through questionnaires and medical records concerning sociodemographic and pregnancy variables: age at partus, body-mass index (BMI), education, employment, comorbidities (migraine, hypertension, diabetes, metabolic disease, allergy, irritable bowel syndrome, alcohol/drug addiction, chronic pain or other disease), history of depression (previous depression or contact with psychiatrist), sleeping habits before pregnancy, domestic violence in present or previous relationship, alcohol consumption, smoking before pregnancy and currently, parity, planned pregnancy, pregnancy complications (vaginal bleeding, contractions, symphysiolysis, gestational diabetes, metabolic disease, hypertension, preeclampsia or other condition (gestational week 32) and fear of delivery.

Out of the 5429 women who accepted the study invitation, 4329 (79.7%) provided full follow-up answers and were included in the current study. Written informed consent was obtained from all study participants.

### Details of Ethics Approval

The study was approved by the Regional Ethical Review Board in Uppsala (reg. no. 2009/171) and was conducted according to the standards of the Declaration of Helsinki.

## Results

### Statistical analysis

The main exposure of the study was NVP and the outcome was depressive symptoms at GW 32 and at six weeks postpartum. Bivariate associations were examined between the main exposure, the outcome, and sociodemographic and pregnancy data. Possible confounders were chosen based on significant associations in the bivariate analyses and previous literature. Covariates associated with both nausea status and depressive symptoms, at *p* < 0.25^25^, were included in multivariable logistic regression models. In models with depressive symptoms at GW 32 and postpartum week six as the outcome, the EPDS score at GW 17 was also inserted in the models to control for a possible state effect of depressive symptoms on nausea symptoms and vice versa. Unadjusted and adjusted odds ratios (ORs) with 95% confidence intervals (CIs) were calculated. Women who did not experience any NVP served as controls (G0) for the three NVP groups mentioned above. As a sensitivity analysis, the same multivariable models were run after excluding women with previous depression. Statistical significance was set at a *p*-value of <0.05.

IBM SPSS Statistics for Windows, version 21.0, was used for the analyses.

### Baseline study characteristics

Of the total cohort, 3493 (80.7%) women experienced NVP. Two thousand six hundred and thirteen (74.8%) women with NVP up to GW 17 did not require medication against it (G1), while 395 (11.3%) women used medication (G2) and 485 (13.9%) women experienced NVP beyond GW 17 (G3). Of these 485 women, 161 (33.2%) used medication. Median gestational week at which NVP symptoms ceased in G3 group was GW 19, compared with GW 13 in G1 and GW 14 in G2, respectively. Four thousand three hundred and twenty-nine women filled in the EPDS questionnaire at GW 17, 3998 at GW 32, and 3677 women returned a completed EPDS questionnaire at six weeks postpartum. The prevalence of self-reported depressive symptoms was 8.8% (379/4329) at GW 17, 9.3% (371/3998) at GW 32 and 12.3% (452/3677) at six weeks postpartum.

Table 1 presents various background characteristics in relation to nausea groups. The majority of the study sample had age at partus between 29–35 years (56.3%). A normal BMI was found in 70% of women. Previous depression or prior contact with psychiatrist was reported by 54.6% of women and 47.5% of the total sample were primiparas. Women from the G1 group had higher education and were more often multiparas, while those from the G2 group were younger, less educated, and reported more frequently comorbidities, previous depression, domestic violence, pregnancy complications and fear of delivery. Concurrent diseases, previous depression, sleep more than eight hours before pregnancy, pregnancy complications, fear of delivery, and multiparity were significantly higher among study subjects with prolonged nausea (G3).

**Table 1:** Sociodemographic and pregnancy characteristics of study subjects by nausea group.

Variable	n	No Nausea GW ≤ 17 (G0)	Nausea without medication ≤ GW 17 (G1)	Nausea with medication ≤ GW 17 (G2)	Nausea > GW 17 (G3)	*p*-value*
**Age (years)**						**0.031**
≤28	(n = 1102)	199 (23.9%)	653 (25.1%)	120 (30.7%)	130 (27.0%)	
29–35	(n = 2427)	470 (56.4%)	1477 (56.8%)	221 (56.5%)	259 (53.8%)	
≥36	(n = 779)	165 (19.8%)	472 (18.1%)	50 (12.8%)	92 (19.1%)	
*Total*	*4308*	*834*	*2602*	*391*	*481*	
**BMI (kg/m**^**2**^**)**						0.075
<18.5	(n = 111)	26 (3.1%)	60 (2.3%)	11 (2.8%)	14 (2.9%)	
18.5–24.9	(n = 3026)	580 (69.9%)	1867 (71.3%)	265 (67.3%)	314 (65.1%)	
25–29.9	(n = 826)	156 (18.8%)	489 (18.7%)	85 (21.6 %)	96 (19.9%)	
>30	(n = 361)	68 (8.2%)	202 (7.7 %)	33 (8.4 %)	58 (12.1%)	
*Total*	*4324*	*830*	*2618*	*394*	*482*	
**Education**						**0.006**
University	(n = 3411)	655 (78.7%)	2099 (80.4%)	289 (73.7%)	368 (76.0%)	
Secondary school	(n = 907)	177 (21.3%)	511 (19.6%)	103 (26.3%)	116 (24.0%)	
*Total*	*4318*	*832*	*2610*	*392*	*484*	
**Employment**						**0.027**
Part/full time studies	(n = 3932)	770 (92.5%)	2386 (91.3%)	345 (88.0%)	431 (89.0%)	
Maternal leave/unempl./sick leave	(n = 390)	62 (7.5%)	228 (8.7%)	47 (12.0%)	53 (11.0%)	
*Total*	*4322*	*832*	*2614*	*392*	*484*	
**Comorbidities^**						**<0.001**
No	(n = 2176)	475 (57.6%)	1312 (50.6%)	170 (43.4%)	219 (45.6%)	
Yes	(n = 2113)	350 (42.4%)	1280 (49.4%)	222 (56.6%)	261 (54.4%)	
*Total*	*4289*	*825*	*2592*	*392*	*480*	
**Prev. depression/contact w/ psych**.						**<0.001**
No	(n = 1959)	460 (55.4%)	1212 (46.4%)	120 (30.5%)	167 (34.6%)	
Yes	(n = 2360)	371 (44.6%)	1399 (53.6%)	274 (69.5%)	316 (65.4%)	
*Total*	*4319*	*831*	*2611*	*394*	*483*	
**Sleeping habits before pregnancy**						**0.034**
<6 hours	(n = 157)	37 (4.5%)	83 (3.1%)	12 (3.1%)	25 (5.2%)	
6–8 hours	(n = 3534)	688 (83.1%)	2156 (83.1%)	312 (80.0%)	378 (78.4%)	
>8 hours	(n = 605)	103 (12.4%)	357 (13.8%)	66 (16.9%)	79 (16.4%)	
*Total*	*4296*	*828*	*2596*	*390*	*482*	
**Domestic violence**						**<0.001**
No	(n = 3707)	729 (89.9%)	2261 (89.5 %)	315 (81.4%)	402 (86.5%)	
Yes	(n = 483)	82 (10.1%)	266 (10.5%)	72 (18.6%)	63 (13.5%)	
*Total*	*4190*	*811*	*2527*	*387*	*465*	
**Alcohol consumption**						0.927
Rarely/Never	(n = 1598)	316 (99.7%)	1001 (99.8%)	130 (100%)	151 (100%)	
At least once a week	(n = 2)	1 (0.3%)	1 (0.1%)	0	0	
More than once a week	(n = 1)	0 (0.0%)	1 (0.1%)	0	0	
*Total*	*1601*	*317*	*1003*	*130*	*151*	
**Smoking before pregnancy**						0.215
Yes	(n = 1380)	259 (31.3%)	836 (32.1%)	142 (36.2%)	143 (29.8%)	
No	(n = 2923)	568 (68.7%)	1768 (67.9%)	250 (63.8%)	337 (70.2%) *480*	
*Total*	*4303*	*827*	*2604*	*392*		
**Cigarette use now**						0.157
Yes	(n = 20)	7 (1.6%)	9 (0.6%)	3 (1.3%)	1 (0.3%)	
No	(n = 2348)	427 (98.4%)	1395 (99.4%)	230 (98.7%)	296 (99.7%)	
*Total*	*2368*	*434*	*1404*	*233*	*297*	
**Parity**						**<0.001**
Primiparous	(n = 1852)	453 (59.4%)	1045 (44.6%)	166 (47.3%)	188 (42.6%)	
Multiparous	(n = 2047)	309 (40.6%)	1300 (55.4%)	185 (52.7%)	253 (57.4%)	
*Total*	*3899*	*762*	*2345*	*351*	*441*	
**Planned pregnancy**						0.448
Yes	(n = 3641)	713 (85.9%)	2198 (84.5%)	328 (83.0%)	402 (83.1%)	
No	(n = 669)	117 (14.1%)	403 (15.5%)	67 (17.0%)	82 (16.9%)	
*Total*	*4310*	*830*	*2601*	*395*	*484*	
**Pregnancy complications**						**<0.001**
No	(n = 1570)	438 (58.6%)	915 (40.7%)	92 (26.5%)	125 (30.6%)	
Yes	(n = 2183)	310 (41.4%)	1334 (59.3%)	255 (73.5%)	284 (69.4%)	
*Total*	*3753*	*748*	*2249*	*347*	*409*	
**Fear of delivery**						**<0.001**
No fear	(n = 3101)	655 (82.7%)	1870 (77.9%)	255 (69.7%)	321 (73.6%)	
Any fear	(n = 892)	137 (17.3%)	529 (22.1%)	111 (30.3%)	115 (26.4%)	
*Total*	*3993*	*792*	*2399*	*366*	*436*	

*Pearson Chi-Square, ^Comorbidities: migraine, hypertension, diabetes, metabolic disease, allergy, irritable bowel syndrome, alcohol/drug addiction, chronic pain or other disease, Pregnancy complications: vaginal bleeding, contractions, symphysiolysis, gestational diabetes, metabolic disease, hypertension, preeclampsia or other condition (gestational week 32).

### Associations between NVP groups and perinatal depressive symptoms

The unadjusted binary logistic regression model showed a positive association with depressive symptoms in all three NVP groups, compared to G0, at GW 17 and 32 and at six weeks postpartum. However, after adjustment for possible confounders, significant associations with postpartum depressive symptoms remained only for G3, compared to G0 (adjusted OR = 1.66; 95% CI 1.1–2.52) (Table 2).

**Table 2:** Logistic regression-derived Odds Ratios (OR) and 95% Confidence Intervals (95% CI) for self-reported depressive symptoms in gestational week (GW) 17 and 32 (cut-off ≥13) and six weeks postpartum (cut-off ≥12) in relation to nausea during pregnancy, unadjusted and adjusted for possible confounders (age, BMI, education, comorbidities (any), previous depression or contact with psychiatrist, sleeping habits before pregnancy, domestic violence, smoking before pregnancy, parity, fear of delivery).

	GW 17		GW 32		Postpartum week 6	
	OR	95% CI	OR	95% CI	OR	95% CI
*Unadjusted*						
Nausea without medication ≤ GW 17 (G1)	1.49	1.08–2.06	1.39	1.02–1.9	1.38	1.03–1.84
Nausea with medication ≤ GW 17 (G2)	2.49	1.65–3.76	2.21	1.47–3.31	2.2	1.5–3.23
Nausea > GW 17 (G3)	2.36	1.59–3.51	1.54	1.02–2.34	2.14	1.49–3.08
	N = 4329		N = 3998		N = 3677	
*Adjusted*						
Nausea without medication ≤ GW 17 (G1)	1.21	0.83–1.75	1.04	0.73–1.5	1.23	0.89–1.69
Nausea with medication ≤ GW 17 (G2)	1.52	0.94–2.45	1.11	0.68–1.81	1.49	0.97–2.3
Nausea > GW 17 (G3)	1.47	0.92–2.36	0.77	0.47–1.28	1.66	1.1–2.52
	N = 3463		N = 3689		N = 3216	

Models with outcome self-reported depressive symptoms at GW 32 and postpartum week six are also adjusted for self-reported depressive symptoms at GW 17. Reference group: no Nausea.

Similarly, after excluding women with history of depression, only participants with prolonged nausea (G3) were at higher odds for depressive symptoms at six weeks postpartum (adjusted OR = 2.26; 95% CI 1.04–4.92) (Table 3).

**Table 3:** Logistic regression-derived Odds Ratios (OR) and 95% Confidence Intervals (95% CI) for self-reported depressive symptoms in gestational week (GW) 17 and 32 (cut-off ≥13) and six weeks postpartum (cut-off ≥12) in relation to nausea and vomiting during pregnancy, unadjusted and adjusted for possible confounders (age, BMI, education, comorbidities (any), sleeping habits before pregnancy, domestic violence, smoking before pregnancy, parity, fear of delivery.

	GW 17		GW 32		Postpartum week 6	
	OR	95% CI	OR	95% CI	OR	95% CI
*Unadjusted*						
Nausea without medication ≤ GW 17 (G1)	0.81	0.43–1.51	1.13	0.58–2.2	1.31	0.78–2.18
Nausea with medication ≤ GW 17 (G2)	1.84	0.73–4.61	2.94	1.21–7.15	0.98	0.36–2.69
Nausea > GW 17 (G3)	1.11	0.42–2.9	0.72	0.2–2.57	2.18	1.09–4.39
	N = 1955		N = 1816		N = 1681	
*Adjusted*						
Nausea without medication ≤ GW 17 (G1)	0.72	0.35–1.47	1.25	0.58–2.71	1.31	0.75–2.32
Nausea with medication ≤ GW 17 (G2)	1.46	0.51–4.15	2.09	0.66–6.67	0.69	0.22–2.22
Nausea > GW 17 (G3)	1.05	0.36–3.1	0.78	0.2–3.09	2.26	1.04–4.92
	N = 1559		N = 1663		N = 1459	

Models with outcome self-reported depressive symptoms at GW 32 and postpartum week six are also adjusted for self-reported depressive symptoms at GW 17 and after the exclusion of women with previous depression. Reference group: no Nausea.

Previous depression was independently associated with depressive symptoms at GW 17 and 32 as well as at postpartum week six.

**Figure 1:**
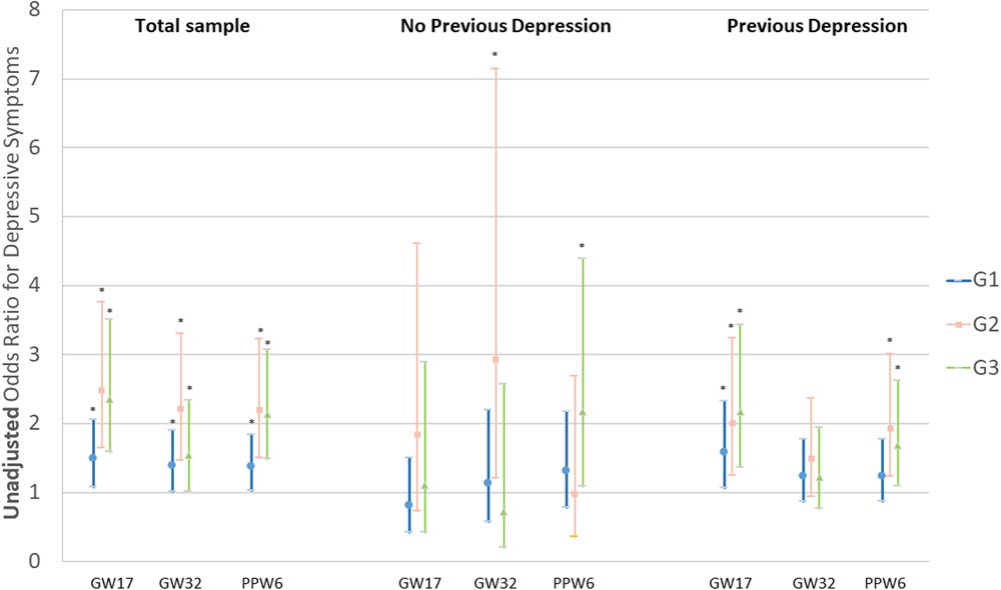
Unadjusted Odds Ratios and 95% Confidence Intervals for depressive symptoms in gestational week (GW) 17, 32 and postpartum week 6 (PPW), in relation to subgroups of nausea and vomiting during pregnancy, in the total sample and women without previous depression and with previous depression. G1: early nausea without medication; G2: early nausea with medication; G3: prolonged nausea; *p* < 0.05.

**Figure 2:**
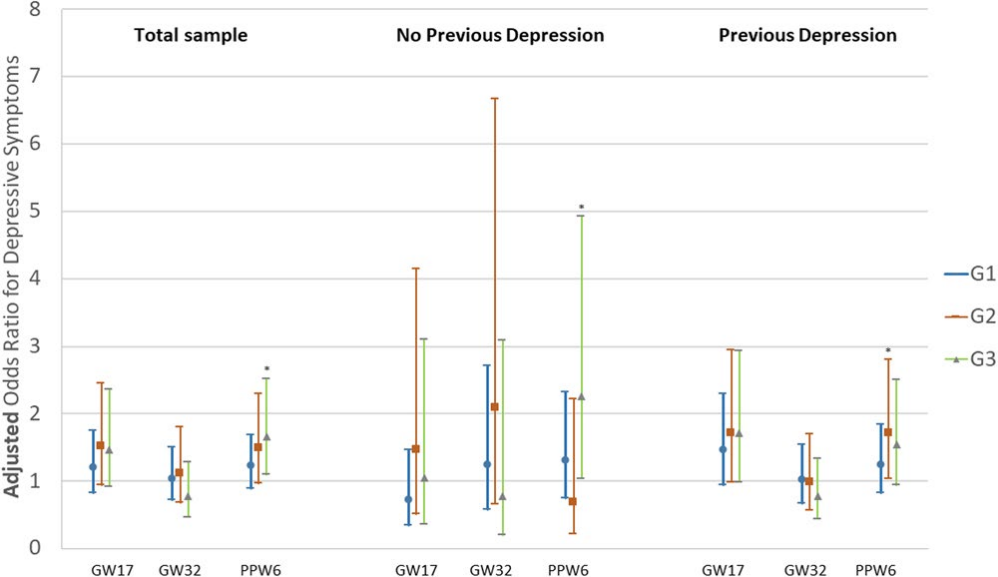
Adjusted Odds Ratios and 95% Confidence Intervals for depressive symptoms in gestational week (GW) 17, 32 and postpartum week 6 (PPW), in relation to subgroups of nausea and vomiting during pregnancy, in the total sample and women without previous depression and with previous depression. G1: early nausea without medication; G2: early nausea with medication; G3: prolonged nausea; *p* < 0.05.

Figures 1 and 2 present unadjusted and adjusted ORs and 95% CI for depressive symptoms in different NVP groups, in the total sample as well as in participants with and without previous depression. In women with previous depression, G2 group was significantly associated with postpartum depressive symptoms, while the association between prolonged nausea (G3) and depressive symptoms was not significant (*p* = 0.086) (Fig. 2). However, an interaction term between previous depression and NVP groups was created and inserted in the regression models for the total sample. Since the association between this term and depressive symptoms was not significant during pregnancy and postpartum, the stratified results in Figs 1 and 2 should be interpreted with caution.

**Figure 3:**
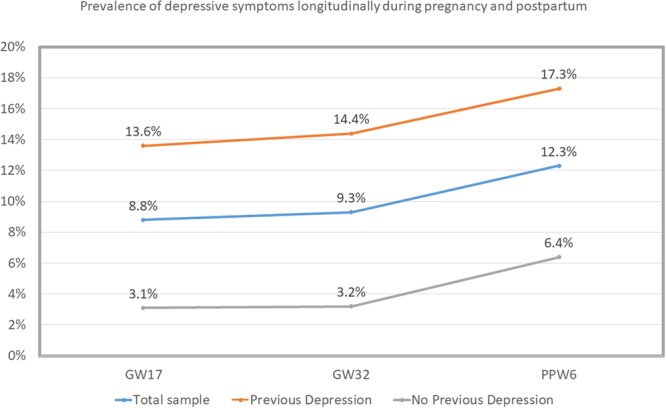
Prevalence of depressive symptoms longitudinally during pregnancy and postpartum, among the three sub-populations (women with previous depression, the total sample and women without previous depression). GW: gestational week, PPW: postpartum week.

Figure 3 shows the prevalence of depressive symptoms among the three previously mentioned sub-populations. As expected, a higher proportion of women with previous depression reported depressive symptoms at all three time-points during pregnancy and postpartum, compared to the total sample and to the women without previous depression (Fig. 3).

## Discussion

The main finding of this study was a positive association between prolonged NVP and self-reported depressive symptoms at six weeks postpartum. Even after the exclusion of women with previous depression, this association remained significant.

Several studies have only focused on hyperemesis gravidarum^13,16,25–27^, whereas others have studied nausea and/or vomiting in general^11,12,28–32^. Consistently with other studies, we found that most pregnant women experienced NVP^2–4^ and about 75% of them belonged to the group G1 (early nausea without medication).

The definition of prolonged nausea is inconsistent, with GW 15 to 20 stated most often as the time point when nausea/HG should diminish^2,33,34^. In the present study, GW 17 was chosen as a discriminating point between early and prolonged nausea, a time-point similar to most other studies. Rates of prolonged nausea vary between 10–45%^4,6,12,35,36^. The prevalence of prolonged nausea in the current study agrees well with the rate of 14.5% reported by Colodro-Conde *et al*.^8^. Moreover, the use of medication against nausea is in good accordance with previous studies^12,37^. Additionally, the prevalence of depressive symptoms in our population is in line with previous studies in high–income countries, reporting figures between 7–30%^38^.

In the present study, a large proportion of women had history of depression or prior contact with psychiatrist (54.6%), a figure higher than in other studies^12,16^. These differences probably can be explained by different methods of assessing and reporting previous depression, as well as a degree of self-selection bias, with women with previous history being more prone to participate in a study focusing on depression. However, it probably did not have an impact on the results. The prevalence of self-reported depressive symptoms during pregnancy in our cohort was 8.8% and 9.3% at GW17 and 32 respectively, which are lower figures compared with a national Swedish sample (13.7–15.7%)^39^. At six weeks postpartum, the figure of 12.3% in the BASIC study is also well comparable with 11.1% prevalence of depressive symptoms two months postpartum in the same sample^39^.

Our findings of an independent association between previous depression and perinatal depressive symptoms confirm the role of previous depression as a strong risk factor for AND and PPD, a finding consistent with previous studies^38,40^.

In a study by Bozzo *et al*., no association was observed between depressive symptoms and nausea, in women without previous depression^11^, a finding contradicting our results that point out NVP as an independent risk factor of PPD even in previously non-depressed women. However, this study only included 57 women and could lack sufficient power. A case-control study on 78 cases and 82 controls by Aksoy *et al*. reported an increased frequency of depressive disorders during pregnancy in women with HG and without history of depression^14^. However, the authors of this study assessed severe hyperemesis with a loss of at least 5% of body weight, the study sample was rather small and they used Beck Depression Inventory for measurement of severity of depression, which might be an inappropriate instrument for NVP patients as highlighted by Fejzo *et al*.^27^. In another study, patients with severe HG in the first trimester, but without prior psychiatric disease, not only presented a negative psychiatric status during pregnancy at the time of HG symptoms, but also had higher risk of PPD^15^. Our results for prolonged nausea are also in line with the study by Meltzer-Brody *et al*. that found an association between severe NVP and PPD^17^. As opposed to the study by Kjeldgaard *et al*.^14^, the present study did not show significant associations between NVP and depression during pregnancy. However, our study population was not restricted to women with HG, since this study intended to examine the whole NVP spectrum and not only women with hyperemesis gravidarum.

The pathophysiology of NVP is multifactorial^41,42^. The dilemma whether NVP and HG are independent diseases is not yet resolved. Twin and family studies provide evidence for strong genetic predisposition in NVP^8,43,44^, which may account for at least 50% of symptom variation, according to the NVP Genetics Consortium^8,43^.

An altered intracellular Ca^2+^ homeostasis has been suggested in the pathophysiology of mood disorders^45^ and genetic analysis of subjects with severe HG has also revealed an association with intracellular calcium release channel RYR2 (Ryanodine Receptor 2) involved in vomiting^46^. It can be hypothesized that prolonged nausea probably has a different aetiology, compared with early types of NVP^47^. It is therefore possible that prolonged NVP may also have a stronger association to perinatal mood disorders. In fact, different trajectory patterns of perinatal depressive symptoms may be indicative of underlying differences in aetiology. Our results support the hypothesis about the heterogeneous pattern of NVP and depressive symptoms during pregnancy and postpartum, considering that our findings regarded only prolonged nausea and were observed regardless of the occurrence of previous depression in the study population. The observed increase of depressive symptoms at six weeks postpartum (Fig. 2) may be explained by a more abrupt hormonal decrease occurring after placental expulsion^48,49^, in combination with an altered immune response. Mullin *et al*. hypothesized that prolonged nausea might be of autoimmune origin^47^. In the present cohort, the women from G3 group reported more frequently comorbidities, including allergy, diabetes and irritable bowel syndrome. It could be speculated that the observed increase of depressive symptoms at six weeks postpartum might reflect these hormonal changes in an altered immunologic environment. Although the association between G3 and postpartum depressive symptoms was not significant, among women with previous depression, this finding still points to the same direction as the rest of our results for women without previous depression and the total sample.

Future research should focus on elucidating different conditions within the spectrum of NVP, with emphasis on the time of onset and ceasing of disease symptoms as well as on the duration of clinical symptoms. In this context, prolonged NVP regardless of severity of symptoms is a particularly interesting condition for further study.

The sample size, the prospective, longitudinal, population-based design and the availability of a large number of background variables can be considered as strengths of this study. Moreover, a validated instrument for the assessment of depression during pregnancy and postpartum period has been used, which can be considered as a study strength since only a few studies have focused on the association between nausea in pregnancy and perinatal depression using validated instruments so far^50^. Considering the heterogeneity of NVP and the relatively scarce literature on the prolonged form of NVP, this study has attempted to contribute to the understanding of this condition by focusing on different clinically relevant subtypes of the disease. The main limitation of the present study is the lack of a validated instrument for assessing severity of nausea in pregnancy, such as the PUQE score^51^. The women’s medical records were not assessed for information about hospitalization for HG and the frequency of vomiting episodes was not known. The present study focused instead on possible associations between different subtypes of NVP, based on the gestational week at which symptoms diminished, the use of medication, and the occurrence of perinatal depressive symptoms. Nevertheless, previous studies have shown that the women’s own perception of nausea severity was not dependent on the severity of physical symptoms^10,18^. As shown in the NVP Genetics Consortium twin study, the genetic correlation between duration and severity of NVP was almost perfect^8^. In the present study, the reported time-point when nausea symptoms ceased was used as a discriminatory factor between early and prolonged NVP, regardless of the severity of symptoms. No data on symptom onset was available, however it can be hypothesized that in the vast majority of women symptoms had already appeared after the first few gestational weeks. This limitation may be at the same time a strength of the study, since it included all women with nausea regardless of severity of clinical symptoms.

The mother study of the present project – the BASIC study – has a relatively low participation rate, which may raise questions about the representability of the cohort. Compared with pregnant women in the study area at the time, participants in the BASIC study had higher education and were more likely to have been born within Scandinavia. However, since women with lower socioeconomic status have a higher risk of depression as well as NVP^52^, an association seems plausible even among non-participants. Even though women with previous depression may have been oversampled because of the recruitment information about the study objective, a corresponding selection bias for NVP is unlikely since the information did not single out NVP among the risk factors of study. Still, the study should preferably be replicated among foreign-born women in Sweden, since some earlier reports indicate different rates of NVP depending on ethnicity^52^.

In conclusion, women with prolonged NVP beyond GW 17 have higher odds for self-reported depressive symptoms at six weeks postpartum, a finding observed even among women without previous depression. Prolonged nausea may have a different pathophysiologic background. Women experiencing prolonged nausea need proper support from health care professionals to reduce the risk of developing PPD.

## Data Availability

The datasets generated during and/or analysed during the current study are available from the corresponding author on reasonable request.
